# Inhibition of p22^phox^ Suppresses Epithelial Ovarian Cancer Cell Proliferation and Tumorigenesis

**DOI:** 10.7150/jca.54163

**Published:** 2021-05-19

**Authors:** Qi Li, Xiaomin Feng, Fengnan Niu, Jun Yang, Yuemei Xu, Xiaohong Pu, Jun Chen, Xiangshan Fan, Binghua Jiang, Qin Huang

**Affiliations:** 1 Department of Pathology, Nanjing Drum Tower Hospital, The Affiliated Hospital of Nanjing University Medical School, Nanjing, China.; 2 Department of Pathology, Affiliated Obstetrics and Gynecology Hospital of Nanjing Medical University/Nanjing Maternal and Child Health Hospital, Nanjing, China.; 3 Institute of Medical and Pharmaceutical Sciences, the Academy of Medical Sciences, Zhengzhou University, Zhengzhou, China.; 4 Department of Pathology and Laboratory Medicine, Veterans Affairs Boston Healthcare System and Harvard Medical School/Brigham and Women's Hospital, West Roxbury, MA, USA.

**Keywords:** p22^phox^, epithelial ovarian cancer, p53, tumorigenesis, chemosensitivity

## Abstract

The aim of this study was to investigate the biological role and molecular mechanism of p22^phox^ in epithelial ovarian cancer. Immunohistochemistry was employed to determine the p22^phox^ expression level in epithelial ovarian cancer tissues. The effects of p22^phox^ on epithelial ovarian cancer cell proliferation, tumorigenesis, and chemosensitivity were evaluated by CCK-8, EdU assay, colony formation and apoptosis assays in vitro and by mouse experiments in vivo. Immunoprecipitation analyses were utilized to explore the potential mechanisms of p22^phox^ mediated downstream signaling, and RT-PCR and western blot were used to confirm the relevance. P22^phox^ expression could be detected in epithelial ovarian cancer tissues and normal fallopian epithelial cells. Silencing p22^phox^ suppressed epithelial ovarian cancer cell proliferation and colony formation capacity *in vitro*, and inhibited the tumor growth in nude mice bearing the A2780 xenograft *in vivo*. Mechanistic investigations showed that p22^phox^ regulated proteasome ubiquitination and subsequent proteasome-dependent degradation of p53 in A2780 and U87 cells *in vitro*. Furthermore, knockdown of p22^phox^ significantly increased the chemosensitivity of A2780 cells to cisplatin or paclitaxel. These results suggested that p22^phox^ as a pivotal oncogene during epithelial ovarian cancer carcinogenesis and p22^phox^ inhibition might be a potential therapeutic strategy for epithelial ovarian cancer.

## Introduction

Epithelial ovarian cancer is the leading cause of female cancer deaths, mainly attributable to the absence of specific early symptoms and effective tools for early detection. As a result, less than one-half of women with epithelial ovarian cancer survive beyond 5 years after diagnosis 1. Epithelial ovarian cancer is a highly heterogeneous tumor and classified many histopathologic types, such as high-grade serous (HGSC), mucinous (MUC), clear cell (CCC), endometrioid (ENOC), and undifferentiated carcinomas 2, 3. Among those carcinoma types, HGSC has been established as of the fallopian tube origin, CCC and ENOC were endometriosis-associated ovarian cancer 4, 5. In general, the prognosis of epithelial ovarian cancer is significantly related to histopathologic types. Therefore, it is critical to illustrate the tumorigenesis mechanism of the occurrence and development of each type of epithelial ovarian cancer to aid in individualized treatment. Unfortunately, it remains unsettled as to the molecular mechanisms of epithelial ovarian cancer.

P22^phox^ is a critical component of the NOX complex_._ Studies have demonstrated that downregulation of p22^phox^ results in decreased activity of several NOX enzymes 6, 7. As a regulatory protein that binds NOX for an effective enzymatic activity, p22^phox^ is associated with several diseases, such as cardiovascular disease and chronic granulomatous disease 8, 9. Moreover, p22^phox^ has been recognized as an oncogene. The study has shown elevated levels of p22^phox^ in primary pancreatic cancer tissues; stable knockdown of p22^phox^ inhibited pancreatic tumor growth 10. In addition, downregulation of p22^phox^ inhibited Akt-dependent phosphorylation of tuberin and stabilized tuberin protein levels in VHL-deficient renal carcinoma cells 11. Furthermore, p22^phox^ has been reported to function as a cisplatin-resistant factor that suppresses DNA adduct-induced apoptosis by blocking cisplatin uptake into the nucleus and activating the phosphatidylinositol 3-kinase (PI3K)/Akt pathway in oral squamous cell carcinoma 12, 13. However, whether the expression of p22^phox^ occurs in the different epithelial ovarian cancer types and the role of p22^phox^ in epithelial ovarian cancer progression has not been established.

In this study, we performed a comprehensive analysis of p22^phox^ expression in the clinical specimens of HGSC, MUC, CCC, ENOC, and undifferentiated carcinomas by IHC. We demonstrate that inhibition of p22^phox^ suppressed the proliferation and tumorigenesis of A2780 and SKOV3 cells both *in vitro* and *in vivo*. Mechanism study reveals that silencing p22^phox^ suppressed proteasome ubiquitination and subsequent proteasome-dependent degradation of p53 in A2780 and U87 cells. Furthermore, p22^phox^ knockdown A2780 cells more sensitive to cisplatin or paclitaxel treatment. In summary, our findings delineated the clinical significance, biological function, and molecular mechanisms of p22^phox^ in epithelial ovarian cancer progression.

## Materials and Methods

### Human Tissue Samples

Human epithelial ovarian cancer samples (63), normal fallopian epithelial tissues (10), normal ovarian tissues (10), and normal endometrial epithelial tissues (10) were obtained from the Department of Pathology, Nanjing Drum Tower Hospital, the Affiliated Hospital of Nanjing University Medical School, China. This study was approved by the hospital institutional review board, and written informed consent was obtained from all patients. The study complied with the principles of the 2008 update of the Declaration of Helsinki.

### Cell Culture and Reagents

Human ovarian cancer cell lines (A2780, SKOV3, HO8910) and normal human ovarian epithelial immortalized cell line (IOSE386) were maintained in RPMI-1640 medium (HyClone, Logan, UT, USA), containing 10% fetal bovine serum (FBS; Gibco, USA) with 100 units/ml penicillin and 100 mg/ml streptomycin. Human OVCAR-3 cells were cultured in RPMI-1640 containing 20% fetal bovine serum with 100 units/ml penicillin and 100 mg/ml streptomycin. Human U87 glioblastoma cells were cultured in DMEM supplemented with 10% FBS, 100 units/ml penicillin, and 100 ng/ml streptomycin. Cells were incubated in an atmosphere with 5% carbon dioxide at 37 °C. Cycloheximide (CHX) was purchased from Beyotime Institute of Biotechnology, Jiangsu, China.

### Immunohistochemistry (IHC)

Representative formalin-fixed paraffin-embedded tissue sections of 63 ovarian tumors in five epithelial ovarian cancer histopathologic types (HGSC, MUC, CCC, ENOC and undifferentiated carcinomas) and 30 normal tissues (ovarian, fallopian tube and endometrioid tissue) were prepared for evaluation of p22^phox^ protein expression by IHC, using an anti-p22^phox^ antibody with the following routine procedure. 5-μm sections were prepared. After dewaxing and hydration, sections were heated in citrate buffer in a microwave oven for 20 min for antigen retrieval. Anti-p22^phox^ rabbit polyclonal antibody (1:100, ab75941, Abcam Company, Cambridge, U.K.) was incubated with the sections overnight at 4 °C. The sections were then washed thrice in PBS for 3 min/wash and subsequently treated with a secondary antibody (ZSGB-BIO, Beijing, China) for one hour at 37 °C, followed by three washes in PBS for 3 min/wash. The p22^phox^ protein expression was visualized with diaminobenzidine (DAB) staining and visualized by light microscopy. A histological score (H-score) was constructed that measures the intensity and distribution of the signal using the formula: [3 (strong signal) × (percentage of cells with strong signal)] + [2 (moderate signal) × (percentage of cells with moderate signal)] + [1 (weak signal) × (percentage of cells with weak signal)]. The histological score ranges from 0 to 300.

### Establishment of Stable Ovarian Cancer Cell Lines

The shp22^phox^ plasmid was obtained from a lentivirus-based human shRNA library (GeneChem, Shanghai, China). Briefly, shp22^phox^ plasmid DNA and the transfection complex DNAs were transfected into human embryonic kidney 293T cells, using the trans-lentiviral packaging kit (Open Biosystems, Shanghai, China) to generate the lentivirus stock. The validated scrambled control plasmid (shSCR) was also transfected into 293T cells as vector control. Lentiviruses in the supernatant were collected and used to transduce A2780 and SKOV3 cells, followed by puromycin selection.

### Antibodies and Immunoblotting

The following antibodies were used in this study: P53 (Cell Signaling Technology, Danvers, MA, USA); GAPDH, Myc-tag, and HA-tag (Bioworld Technology, Atlanta, Georgia, USA). Western immunoblot analysis was performed with the whole-cell lysates in 12% sodium dodecyl sulfate-polyacrylamide gel electrophoresis (SDS-PAGE). The resolved proteins were transferred into polyvinylidene fluoride (PVDF) membrane (Roche, Switzerland), followed by incubation with specific antibodies against different indicated protein factors and treated with secondary antibody (Bioworld Technology, Atlanta, Georgia, USA). Immunoreactivity was visualized by enhanced chemiluminescence (Thermo Fisher Scientific Cat. Rockford, IL, USA).

### RNA Extraction, Reverse Transcription PCR, and Quantitative Real-Time PCR

Total RNA was isolated from harvested cells with the Trizol reagent (Invitrogen, CA, USA), according to the manufacturer's instructions. The mRNA levels of p22^phox^ and p53 were determined by reverse transcription of total RNAs using oligo(dT) primer and the PrimeScript RT Reagent Kit (Takara, Dalian, China), according to the manufacturer's instructions. The cDNAs were amplified by qRT-PCR using the SYBR Premix DimerEraser kit (Takara, Dalian, China) on the 7900HT system, according to the manufacturer's instructions. Fold changes were calculated by relative quantification (2^-ΔΔCt^). The expression of GAPDH was used as an endogenous control. The following p53 primer sequences were used for real-time PCR: p53F: 5′-GCC TGA GGT TGG CTC TGA-3′; p53R: 5′-GTG GTG AGG CTC CCC TTT-3′. The p22^phox^ and GAPDH primers were used as previously described 14.

### Immunoprecipitation

A2780 cells were transfected with pCMV6-MYC-p22^phox^ (OriGene Technologies, Rockville, USA) and the ubiquitin recombinant vector, HA-Ub, as indicated in the Lipofectamine 2000 (Invitrogen, Carlsbad, USA) protocol, according to the manufacturer's instructions. At 48 h after transfection, the cells were treated with the proteasome inhibitor, MG-132 (10 μM) (Beyotime Institute of Biotechnology, Jiangsu, China), for 6 h. Whole-cell lysates were prepared by cell extraction and centrifugation (12,000 rpm for 20 min), and protein concentration was determined with the bicinchoninic acid (BCA) assay (Beyotime Institute of Biotechnology, Jiangsu, China). The precleared soluble supernatant was mixed with a polyclonal anti-p53 antibody and incubated overnight at 4 °C. Protein A/G beads were added to the reaction mixture. After washing with PBS, the immune complexes were resuspended in the SDS sample buffer, fractionated by SDS-PAGE, and analyzed by western blotting with a monoclonal antibody against HA.

### Measurement of Cell Proliferation

Cell viability was assessed with the cell counting kit-8 (CCK-8; Dojindo Laboratories, Kumamoto, Japan), according to the manufacturer's instructions. The cell proliferation curves were plotted with the absorbance values against different time points, using the average data from three separate experiments.

EdU immunofluorescence staining was performed for fluorescence detection of replicating DNA, using the EdU kit (Ruibo Biotechnology, Guangzhou, China). Briefly, cells were grown on coverslips until they reached about 50% confluency. The EdU labeling medium was then added, and the cells were incubated for about 60 min. The cells were then fixed with 4% paraformaldehyde and stained with fresh Apollo reaction cocktail. Subsequently, cells were mounted by adding DAPI (Beyotime Institute of Biotechnology, Jiangsu, China). Images were obtained by confocal microscopy Zeiss LSM710 (ZEISS, Germany).

### Soft agar colony formation assay

SKOV3 and A2780 cells, which stably expressed p22^phox^ shRNA or shSCR, were added to growth medium with 0.6% agar and layered onto 1.2% agar beds in six-well plates. The cell culture media was exchanged every 3 days. The colonies were stained with 0.01% crystal violet and counted in 3 weeks. The results were observed under an optical microscope. The experiment was performed in triplicate.

### DNA isolation

Genomic DNA was obtained from IOSE386, OVCAR3, A2780, HO8910, and SKOV3 cell lines using the Genomic DNA Extraction kit (Takara, Dalian, China) according to the manufacturer's instructions. The concentration of extracted DNA was measured using a NanoDrop 2000 spectrophotometer (Thermo Fisher Scientific Cat. Rockford, IL, USA). The targeted fragment was amplified using the PCR-kit according to the instructions of the manufacturer (Takara, Dalian, China). Amplified samples were subjected to agarose electrophoresis with ethidium bromide as the fluorescent dye.

### *In vitro* Chemosensitivity Assay

A2780 cells were seeded at the density of 4,000 cells per well in a 96-well plate overnight. Freshly prepared cisplatin or paclitaxel (Selleck Chemicals, Houston, TX, USA) was added at varying final concentrations ranging from 0 to 80 μM or 0 to 320 nM. At 72 h after treatment, cell viability was assayed using the CCK8 kit.

### Apoptosis Assessment

Flow cytometry was employed to evaluate cellular apoptosis. Cultured cells were harvested, centrifuged, and then resuspended in 100 μl solution containing 2 μl Annexin V-FITC reagent in propidium iodide solution, and then incubated for 15 min at room temperature in the dark. The samples were analyzed by flow cytometry (FACS Canto II, BD Biosciences, USA) within 1 h. Three experiments were performed in triplicate. The data were analyzed with the FlowJo software.

### Tumorigenesis in Nude Mice

Female BALB/c nude mice (6 weeks old) were obtained from the Shanghai Laboratory Animal Center (Chinese Academy of Sciences, Shanghai, China) and maintained in the special pathogen-free environment for one week. Animal handling and experimental procedures followed those described in the Guide for the Care and Use of Laboratory Animals and approved by the Animal Experimental Ethics Committee of Nanjing University. A2780 shp22^phox^ cells or A2780 shSCR cells (5 × 10^6^ cells in 100 μl) were subcutaneously injected into the left flank of nude mice. Tumor sizes were measured with a vernier caliper every 2 days until the tumors were apparent grossly. The tumor volume was calculated with the following formula: volume = 0.5 × Length × Width^2^. After 25 days of implantation, mice were sacrificed and tumors were dissected, formalin-fixed, paraffin-embedded, and sectioned at 5 μm for Ki67 (ZSGB-BIO, Beijing, China) IHC staining were performed as previously described.

### Statistical Analysis

All experiments were repeated three times and the data were averaged with GraphPad Prism 5 (La Jolla, CA, USA) as the mean ±standard deviation (SD). Statistical Analysis Significant difference of means between two groups was examined using the t-test. The difference with P < 0.05 was considered statistically significant.

## Results

### IHC staining of p22^phox^ in epithelial ovarian cancer tissues

**Figure 1:**
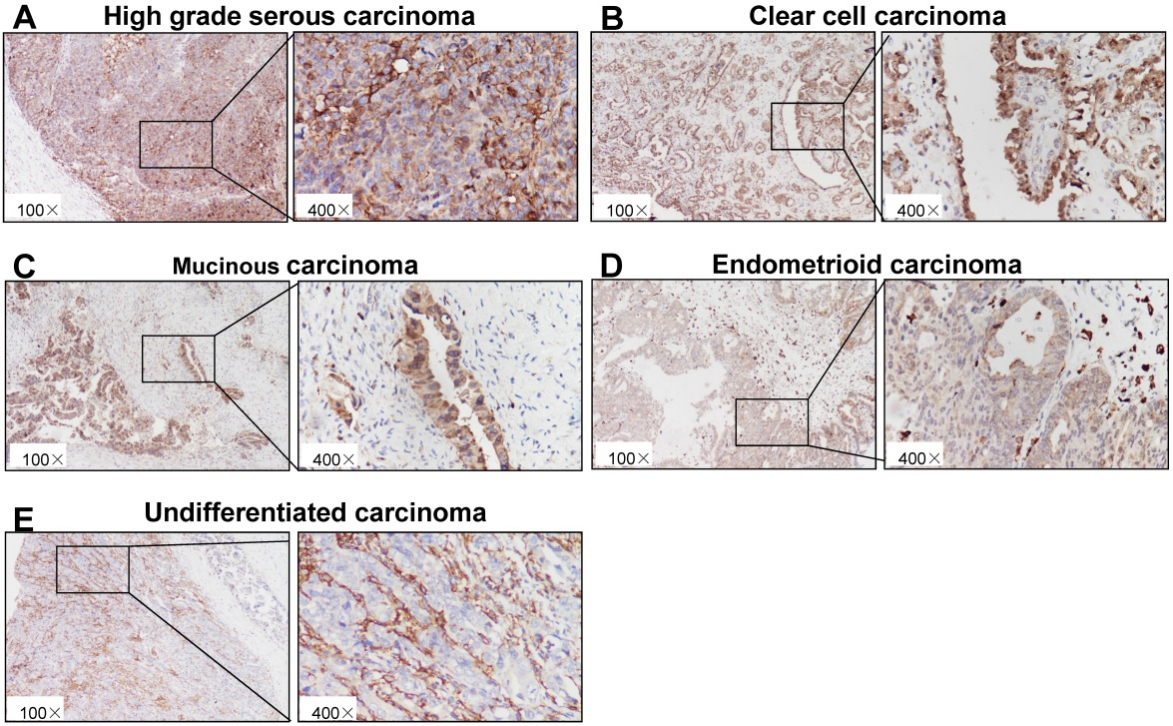
Immunohistochemical staining of p22^phox^ in ovarian cancer tissues. Paraffin-embedded sections of five types of epithelial ovarian carcinoma were stained by immunohistochemistry using an anti-p22^phox^ antibody. The brown color indicates the presence of p22^phox^ expression. Tissues were imaged at a magnification of ×100. Increased magnification (×400) of the area shown in the black box. A, High-grade serous carcinoma. B, Clear cell carcinoma. C, Mucinous carcinoma. D, Endometrioid carcinoma. E, Undifferentiated carcinoma.

As shown in Fig. 1A-E, immunohistochemical staining showed that p22^phox^ expression could be detected in epithelial ovarian cancer tissues with a variety among patients. In the cohort, the average value of p22^phox^ expression in the HGSC is 232.3 ± 30.97, which is the highest among the five types of ovarian carcinomas (HGSC, MUC,CCC, ENOC and undifferentiated ovarian carcinoma) (Table 1).

### P22^phox^ expression in ovarian, fallopian epithelial, endometrial, immortalized ovarian cell and ovarian cancer cells

**Figure 2:**
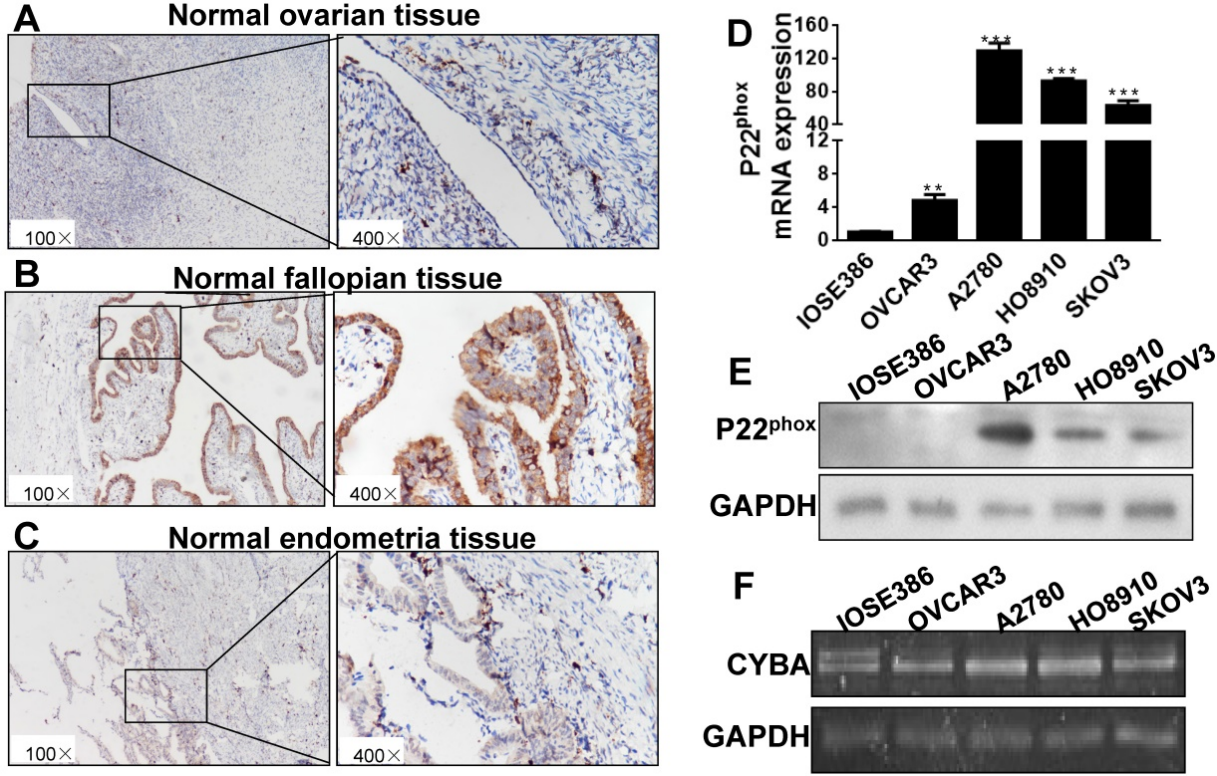
P22^phox^ expression in normal tissues and cells of immortalized and ovarian cancer. A, B, C, Paraffin-embedded sections of ovarian, fallopian epithelial, and endometrial tissues were stained immunohistochemically with an anti-p22^phox^ antibody. D, E, F: Expression levels of p22^phox^ mRNA, protein and DNA in cells of IOSE386, OVCAR3, A2780, HO8910, and SKOV3. ** Indicates a significant difference at P < 0.01, *** indicates a significant difference at P < 0.001.

Mounting clinicopathologic and molecular genetic data have demonstrated various origins of ovarian carcinomas among the different histologic types. For instance, HGSC has been established as of the fallopian tube origin, but both CCC and ENOC develop from the endometrioid tissue 15. As shown in Fig. 2A-C, p22^phox^ immunostaining was intense and diffuse in normal fallopian epithelial cells, but much weaker in normal ovarian and endometrial epithelial cells. In normal ovarian, fallopian epithelial and endometrium tissues, the average value of p22^phox^ expression is 28.3±12.39, 262.3±21.41, 109.0±16.63, respectively (Table 1).

We next investigated the biological consequences of p22^phox^ expression in four human ovarian cancer cell lines, OVCAR3, A2780, HO8910, and SKOV3, and the immortalized cell line, IOSE386. Compared with IOSE386 cells, A2780, HO8910, and SKOV3 cell lines expressed significantly higher levels of p22^phox^ mRNA and protein (Fig. 2D, E) under the condition of the absence of significant differences in the DNA level among all cells tested (Figure 2F).

### Decreased levels of p22^phox^ in A2780 and SKOV3 cells suppressed cell proliferation and clone formation* in vitro*

**Figure 3:**
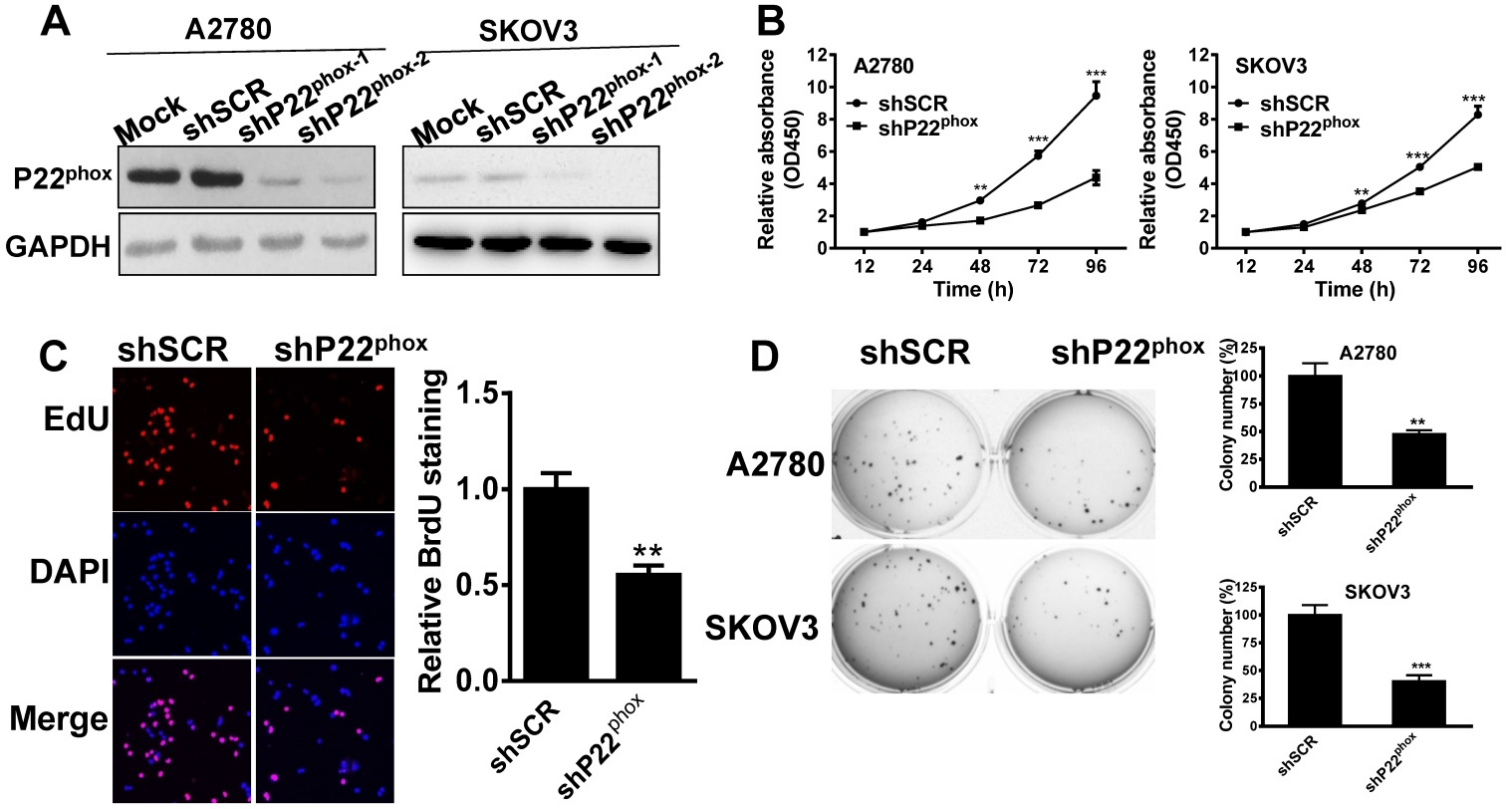
P22^phox^ arrested A2780 and SKOV3 cell growth* in vitro*. A, A2780 and SKOV3 cells stably expressing p22^phox^ shRNA or shSCR were assayed for protein expression of p22^phox^ by western blotting. B, P22^phox^ knockdown arrested cell proliferation in A2780 and SKOV3 cells. Data were presented as mean ± SD from three independent experiments (**P < 0.01, ***P < 0.001). C, With or without p22^phox^ knockdown in A2780 cells were seeded onto coverslips, and cell proliferation was assessed using EdU immunofluorescence staining. The graph on the right shows the percentage of EdU-positive nuclei. The data shown represent the mean of three independent experiments (**P < 0.01). D, Colony formation assays were performed, as described in materials and methods. Colonies were visualized by staining with crystal violet-blue. The relative colony area obtained from three independent experiments is plotted. Data represents the mean ± SD from three independent experiments (**P < 0.01, ***P < 0.001).

We then generated a stable knockdown of p22^phox^ in A2780 and SKOV3 cells and confirmed the inhibition efficiency by western immunoblot analysis (Fig. 3A). Undoubtedly, shp22^phox-2^ was most efficient in suppressing p22^phox^ expression; thus, we used this stable cell line in subsequent experiments. Likewise, the p22^phox^ knockdown A2780 and SKOV3 cell lines exhibited a strong inhibitory effect on cell proliferation, as indicated by the CCK-8 proliferation assay, compared to the shSCR cells (Fig. 3B). Proliferation was also investigated with EdU immunofluorescence staining. As shown in Fig. 3C, the p22^phox^ knockdown cell lines showed a significantly lower rate of proliferation than their shSCR cell line counterparts. A reduced soft agar colony formation capability was also observed in p22^phox^ knockdown cell lines (Fig. 3D). Together, these results demonstrate that p22^phox^ knockdown arrested A2780 and SKOV3 cell growth* in vitro*.

### Downregulation of p22^phox^ in A2780 cells suppressed tumor growth *in vivo*

**Figure 4:**
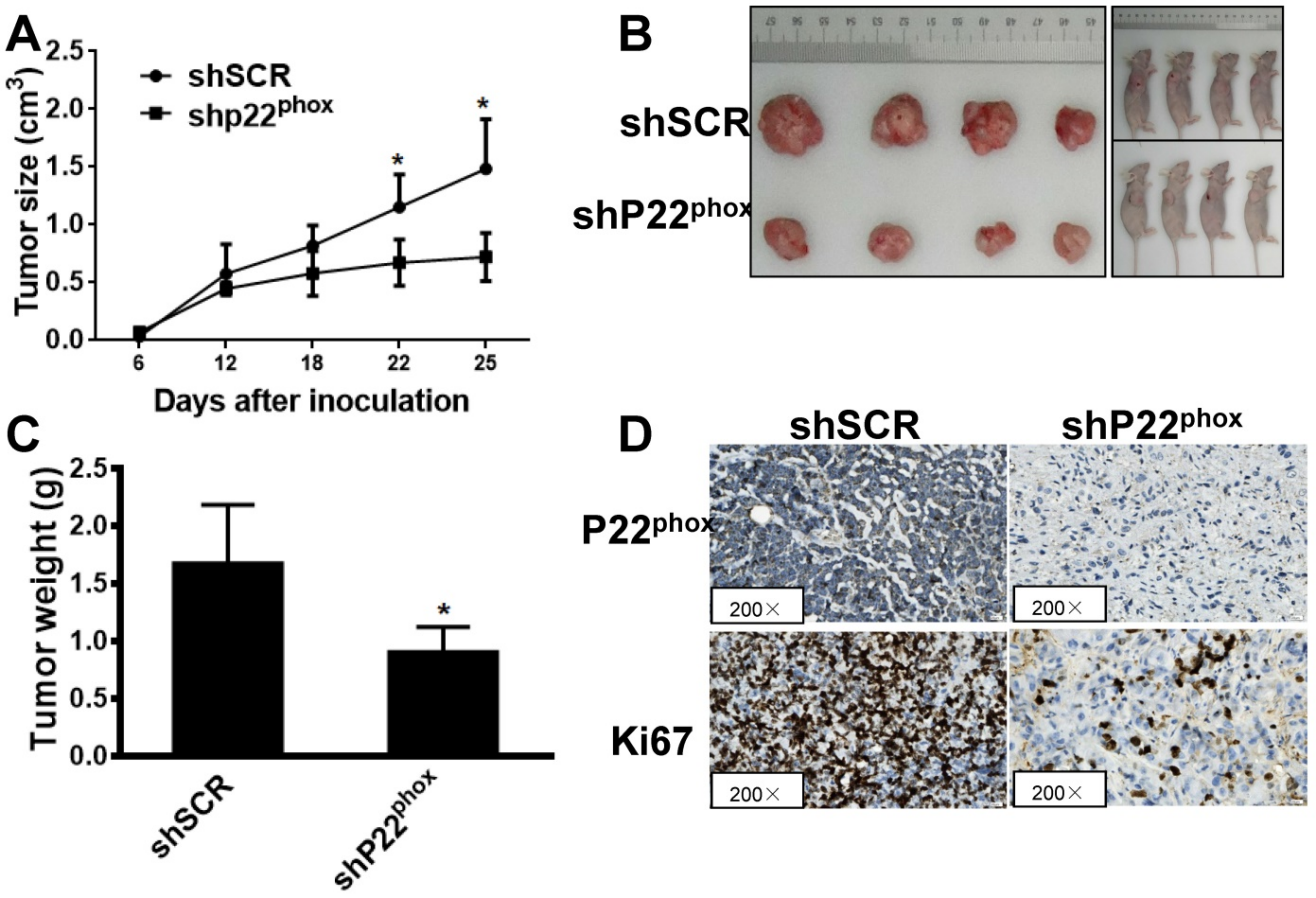
Downregulation of p22^phox^ in A2780 cell inhibited tumor growth* in vivo*. A, B, C, Effect of p22^phox^ on the growth of A2780 cells inoculated into nude mice. Female BALB/c nude mice were subcutaneously injected with 5 × 10^6^ A2780 cells, stably expressing p22^phox^ shRNA or shSCR. Tumor volume and weight were monitored over time, as indicated, and the tumor was excised and weighed after 25 days of inoculation. p22^phox^ knockdown resulted in a decrease in tumor volume and weight (*P < 0.05). D, Expression levels of p22^phox^ and ki67 were analyzed in tumor tissues by immunohistochemistry with representative images shown. Magnification, ×200.

We further clarified the effect of p22^phox^ on tumor growth *in vivo* by subcutaneous injection of the A2780-shp22^phox^ or A2780-shSCR cells into the left flank of nude mice. After 25 days of implantation, we found that the tumor growth rate was significantly slower in the mice injected with A2780-shp22^phox^ cells than in the mice injected with A2780-shSCR cells (Fig. 4A), as revealed by their smaller gross appearance and tumor size (Fig. 4B). We observed that the weight of the A2780-shp22^phox^ cell group was significantly smaller than that of the A2780-shSCR group (Fig. 4C). Likewise, increased ki67 immunoreactivity, which is a measure of rapid cell division, was significantly lower in the tumors from the A2780-shp22^phox^ group than in those from the control group (Fig. 4D). The results suggest that the inhibition of p22^phox^ indeed suppressed A2780 tumor growth *in vivo*.

### Increased levels of p22^phox^ enhanced the ubiquitination and degradation of p53

**Figure 5:**
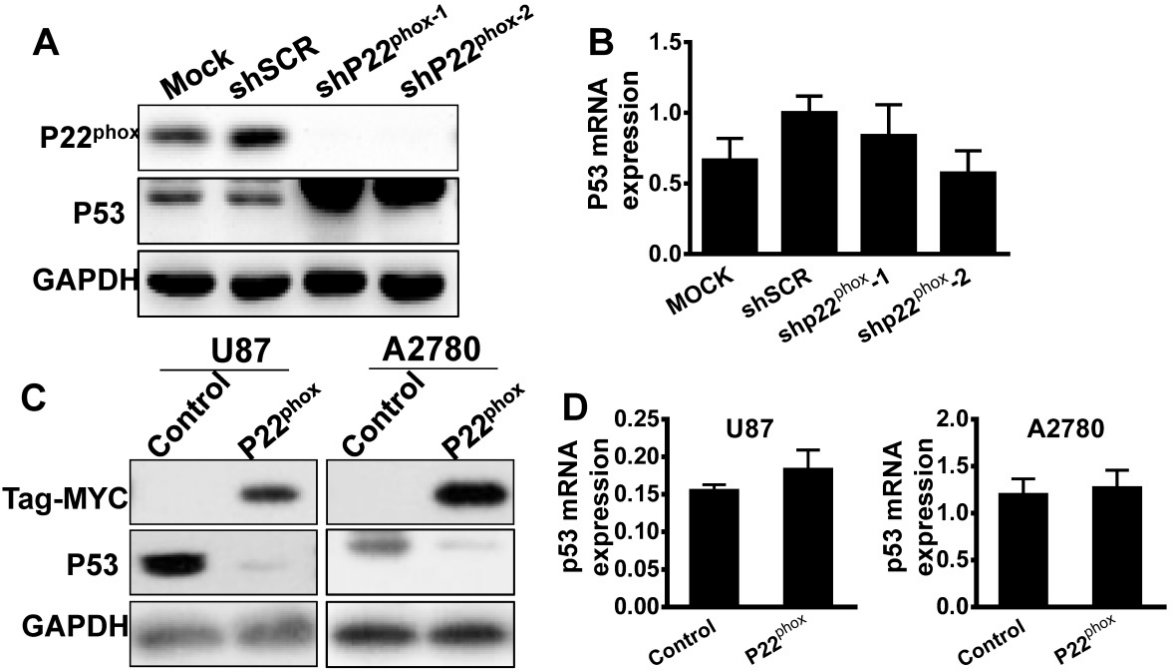

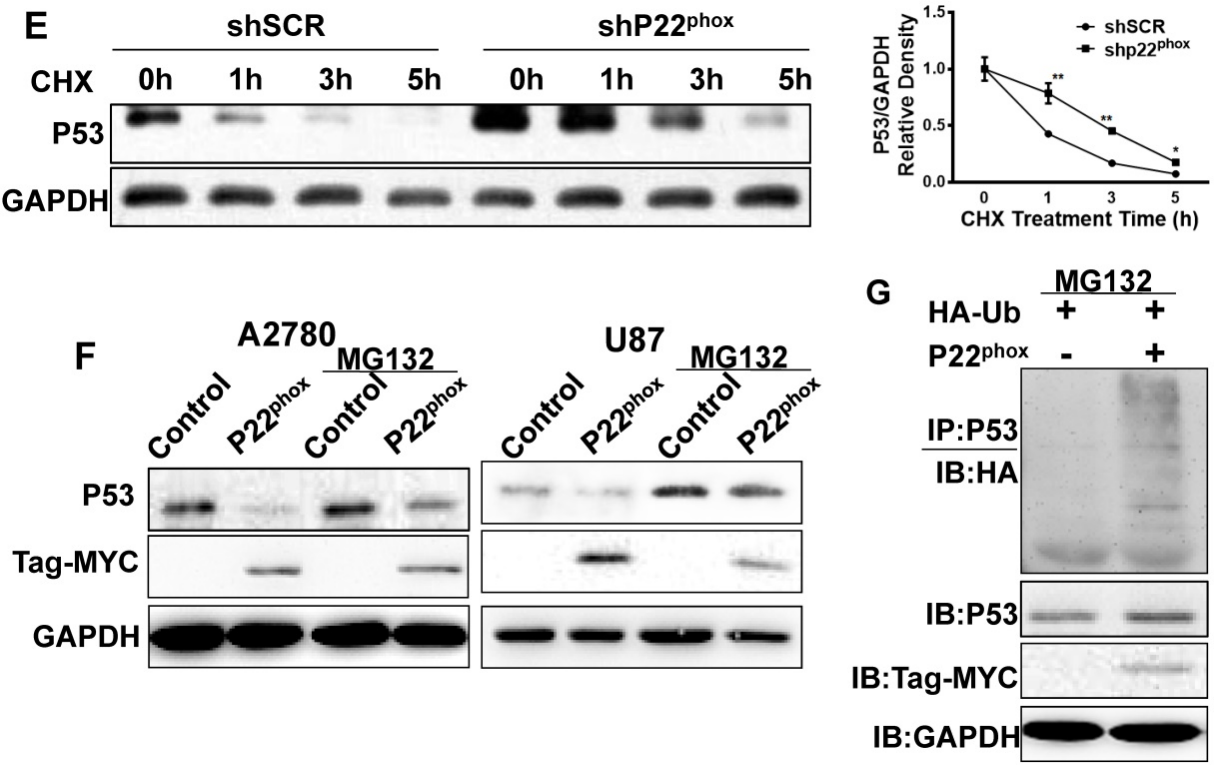
P22^phox^ enhanced the proteasome-dependent degradation of p53. A, B, A2780 cells stably expressing p22^phox^ shRNA or shSCR were assayed for protein and mRNA expression of p22^phox^ and p53 by western blotting and qPCR. Data represent the mean ± SD from three independent experiments (**P < 0.01). C, D, P53 protein and mRNA expression was assessed by immunoblotting and qPCR in A2780 and U87 cells transfected with pCMV6 control or pCMV6-MYC-p22^phox^ expressing vector. E, A2780-shSCR or A2780-shp22^phox^ cells were treated with chlorhexidine (CHX; 50 µg/ml) for the indicated time, and p53 expression was determined by performing immunoblotting (left). The intensity of endogenous p53 at each time point was quantified by performing densitometry (right). Representative results are shown from triplicated experiments with similar results. Values are presented as the mean ± SD, *P < 0.05, **P < 0.01 vs. the control. F, A2780 and U87 cells were transfected with or without the pCMV6-MYC-p22^phox^ expressing vector for 48 h and treated with MG132 for 6 h before harvesting. The cell lysates were immunoblotted with p53. G, A2780 cells were transfected with HA-Ub and with or without pCMV6-MYC-p22^phox^ plasmids for 48 h and treated with MG132 for 6 h before harvesting. The cell lysates were immunoprecipitated with anti-p53 antibodies followed by immunoblotting with anti-HA antibodies. P22^phox^ of input was detected with the anti-MYC antibody.

It is known that the wild type p53 exists in the epithelial ovarian cancer subtypes of ENOC and CCC 16. Immunohistochemistry analysis showed a wild type p53 pattern of expression for p53 in our ENOC and CCC tissues (Figure S1). The A2780 cell line, which is wild type p53, demonstrated a very flat gene copy-number profile with mutations frequently found in ENOC and CCC, such as ARID1A, BRAF, PIK3CA, and PTEN, as reported previously 17. Thus, we investigated p53 expression levels in A2780-shp22^phox^ and A2780-shSCR cells. Interestingly, compared to the A2780-shSCR cell, the A2780-shp22^phox^ cell line showed significant upregulation of p53 protein levels, whereas no significant differences in p53 mRNA levels (Fig. 5A, B). Moreover, with exogenously overexpressed p22^phox^, the p53 protein levels were significantly downregulated compared with the control counterparts (Fig. 5C). Nonetheless, p53 mRNA levels did not change (Fig. 5D). Similarly, in the U87 (wild type p53) cell line with overexpressed p22^phox^, the protein amount, not mRNA levels of p53, was decreased when compared with its control cell line (Fig. 5C, D). Therefore, with those results, we hypothesized that p53 is modulated by p22^phox^ through post-transcriptional or post-translational modifications.

To further investigate the findings that p22^phox^ affected p53 stability, we treated A2780-shp22^phox^ and A2780-shSCR cell lines with cycloheximide (CHX) and examined p53 expression levels. The results demonstrated that p53 stability was enhanced in A2780-shp22^phox^ cells, as compared to A2780-shSCR cells (Fig. 5E). Moreover, overexpression of p22^phox^ increased the amounts of p53 in the presence of the proteasome inhibitor MG132, suggesting the involvement of the ubiquitin chains (Fig. 5F). Accordingly, we verified the existence of p22^phox^-linked p53 ubiquitination in the A2780 cell line by transfecting this cell line with HA-Ub in the absence or presence of p22^phox^. The cell lysates were immunoprecipitated with anti-p53 antibodies, followed by immunoblotting (IB) with an anti-HA antibody. The result demonstrated that overexpression of p22^phox^ markedly promoted the ubiquitination of endogenous p53 (Fig. 5G). Together, these results indicate that p22^phox^ either ubiquitinates p53 or promotes p53 ubiquitination in A2780 cells.

### Diminished expression of p22^phox^ improved the chemosensitivity of A2780 cells to cisplatin or paclitaxel *in vitro*

**Figure 6:**
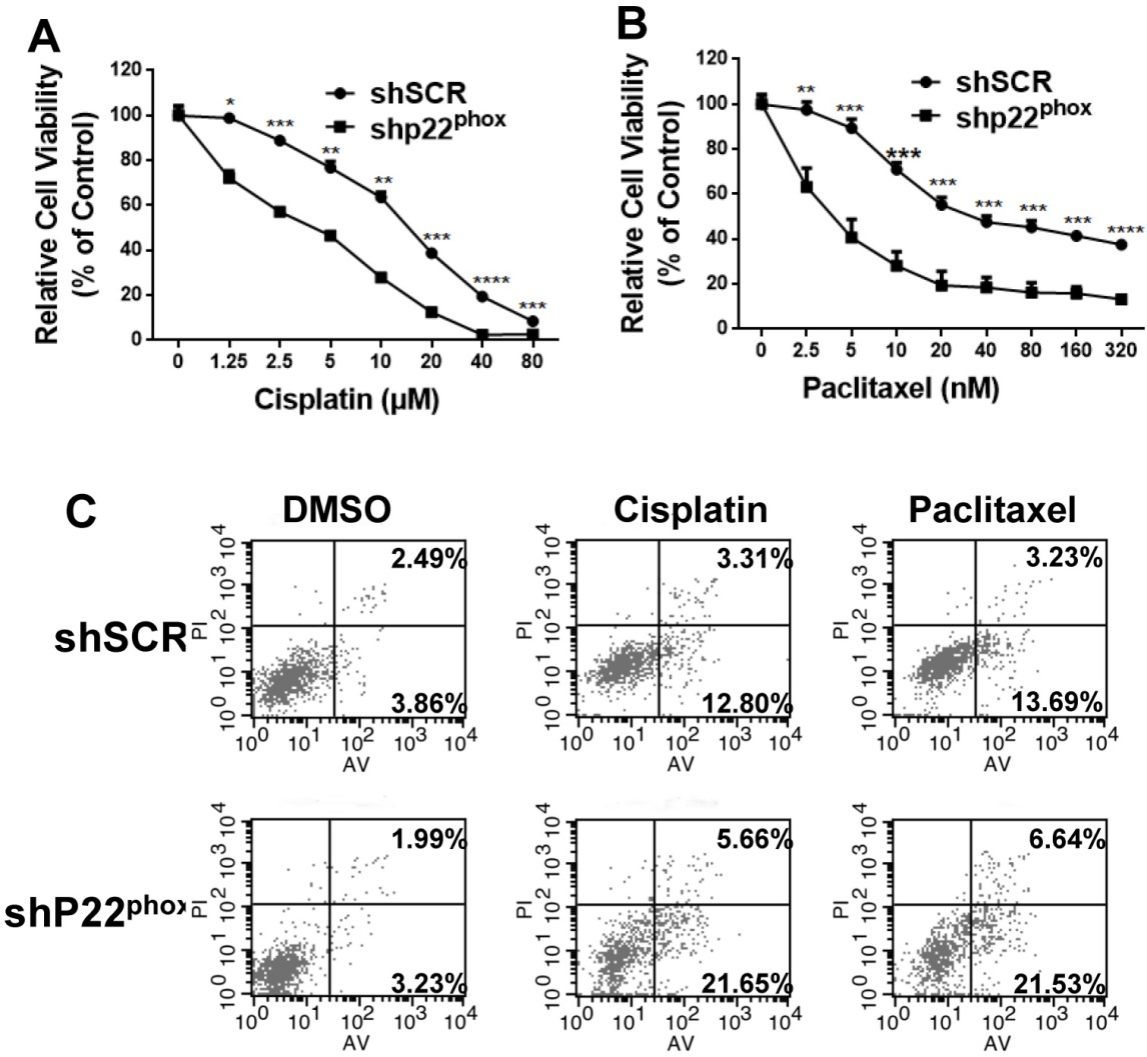
p22^phox^ knockdown increased chemosensitivity of A2780 cells to cisplatin and paclitaxel treatment. A, B, Cell proliferation was evaluated in A2780-shSCR or A2780-shp22^phox^ cells, with or without cisplatin or paclitaxel treatments at different doses. The CCK-8 assay was performed 72 h after treatment. *, **, ***, and **** indicate significant difference at P < 0.05, P < 0.01, P < 0.001, and P < 0.0001, respectively. C, A2780-shSCR or A2780-shp22^phox^ cells were pretreated with or without 10 µm cisplatin or 20 nm paclitaxel for 72 h and then subjected to apoptosis analysis by flow cytometry.

To explore the potential effects of p22^phox^ on chemosensitivity to cisplatin or paclitaxel, we treated A2780-shp22^phox^ and A2780-shSCR cells with different concentrations of cisplatin or paclitaxel. As shown in Fig. 6A and 6B, A2780-shp22^phox^ cells significantly decreased chemosensitivity to cisplatin or paclitaxel *in vitro*; the cell viability was also considerably suppressed, as concentrations of cisplatin (range = 0-80 μΜ), or paclitaxel (range = 0-320 nΜ) were increased, compared to A2780-shSCR cells. This enhanced chemosensitivity to cisplatin or paclitaxel treatment was subsequently investigated by flow cytometry to assess the rate of cellular apoptosis. We found that the combination of p22^phox^ knockdown and cisplatin or paclitaxel treatment significantly increased cell apoptosis rates in the A2780 cell line (Fig. 6C). These results suggest that the downregulation of p22^phox^ expression rendered the ovarian A2780 cells more sensitive to cisplatin or paclitaxel treatment.

## Discussion

In the present study, immunohistochemical staining showed that p22^phox^ expression could be detected in HGSC, MUC, CCC and ENOC tissues with a variety among patients. The p22^phox^ expression value in the HGSC is the highest among the five types of ovarian carcinomas. This finding is consistent with that reported previously, high grade serous carcinoma accounts for majority of the cases and most of the lethality 15. Epithelial ovarian cancer is hypoxic by nature and could grow under hypoxic conditions, in which several cellular signaling pathways are known to be activated to enhance tumor cell proliferation and metastatic capacity 18, 19. Until recently, it was reported that p22^phox^ is ubiquitinylated under normoxic conditions and that this modification is decreased under hypoxia, indicating that p22^phox^ is stabilized and accumulates under hypoxia 20. It is, thus, plausible that p22^phox^ overexpression could result from the hypoxic microenvironment in ovarian tumors. Further experiments are needed to prove this relevant speculation.

Mounting clinicopathologic and molecular genetic data have demonstrated HGSC has been established as of the fallopian tube origin, but both CCC and ENOC develop from the endometrioid tissue 15. The H-score of p22^phox^ in normal fallopian and HGSC tissues was 262.3±21.41, 232.3±30.97, respectively. The high expression of p22^phox^ in the HGSC and fallopian epithelial indirectly suggests that the lesion originates from the tubal epithelium. In normal endometrium tissue, CCC and ENOC, the average value of p22^phox^ expression was 109.0±16.63, 207.3±29.75 and 180.7±61.43, respectively. Higher expression of p22^phox^ in CCC and ENOC tissues than in the normal endometrial epithelium suggests that p22^phox^ might promote the occurrence and development of those two ovarian carcinomas. Additionally, our results confirmed that p22^phox^ knockdown decreased A2780 and SKOV3 cell proliferation and soft agar colony formation capacity. Furthermore, tumor growths in mouse xenograft models were inhibited by p22^phox^ knockdown, further confirming that p22^phox^ acts as oncogenes in epithelial ovarian cancer cells.

P22^phox^ is a critical component of the NOX complex, which is involved in the regulation of endogenous ROS level 6, 7. P53 protein is the core of a network of pathways in which ROS play critical roles, and is an essential transcriptional factor regulating cellular homeostasis 21, 22. It is well known that the CCC and ENOC possess the wild type p53, we found that inhibition p22^phox^ appeared to upregulate p53 protein levels, while no significant reduction of p53 mRNA levels in A2780 cells. These results motivated us to hypothesize that p22^phox^ was involved in p53 protein polyubiquitination and proteasome-dependent degradation. Further, we verified the hypothesis by performing *in vitro* ubiquitination studies in A2780 and U87 cells. MDM2, a ubiquitin ligase for p53, plays a central role in the regulation of the stability of p53 23. Moreover, Pirh2 and LIF could also play roles in regulating this process 24, 25. Additionally, the deubiquitinating enzyme Otubain and the ubiquitin specific protease-7 (USP7) can remove the ubiquitin from P53 26, 27. Furthermore, p53 is involved in apoptosis, cell cycle regulation, and DNA replication and repair, and regulates the expression of many genes, including PUMA, NOXA, FAS, and p21 genes. The exact pathways of p53 involving p22^phox^ in A2780 and U87 cells require further verification to gain more insight into the mechanism.

Cis-diamminedichloroplatinum (II) (cisplatin II) and paclitaxel are widely used for treating various solid tumors, including cervical, ovarian, and breast cancers. It is inevitable to develop chemotherapeutic drug resistance after a prolonged period of usage. To date, cisplatin- or paclitaxel-based chemotherapy, combined with other chemotherapeutic agents, remains the first-line treatment for ovarian cancers 28. Recent reports have shown that p22^phox^ is a cisplatin-resistant gene that suppresses DNA adduct-induced apoptosis by blocking cisplatin uptake into the nucleus and activating the PI3K/Akt pathway 13. Our study found that interfering with p22^phox^ function could enhance the drug sensitivity of ovarian cancer cells to cisplatin or paclitaxel and thus provides a new concept for the treatment of ovarian cancer.

Herein, we provide the evidence that p22^phox^ expression could be detected in epithelial ovarian cancer tissues. Inhibition of p22^phox^ showed the profound effects in retarding ovarian cancer cell proliferation and tumorigenesis *in vitro* and *in vivo*, complementing its biological function via influencing the proteasome ubiquitination and subsequent proteasome-dependent degradation of p53. Moreover, downregulation of excessive expression of p22^phox^ significantly increased the chemosensitivity of A2780 cells to cisplatin or paclitaxel treatment. Overall, our findings define a useful treatment target for combating epithelial ovarian cancer growth.

## Supplementary Material

Supplementary figure S1.

## Figures and Tables

**Table 1:** Immunohistochemical analysis of p22^phox^ in epithelial ovarian carcinoma and normal tissues

	N	Descriptive statistics (H-SCORE)	
		mean±SD	min-max
**Epithelial Ovarian Carcinoma**			
High grade serous carcinoma	45	232.3±30.97	157-275
Clear cell carcinoma	6	207.3±29.75	173-243
Mucinous carcinoma	4	207.3±28.5	168-232
Endometrioid carcinoma	7	180.7±61.43	58-249
Undifferentiated carcinoma	1		
**Normal tissues**			
Ovarian	10	28.3±12.39	10-49
Fallopian epithelial	10	262.3±21.41	212-293
Endometrium	10	109.0±16.63	90-140
