# Supplementary material for: Inhibition of p22phox Suppresses Epithelial Ovarian Cancer Cell Proliferation and Tumorigenesis
Source: J Cancer. 2021 May 19;12(14):4277–87. doi: 10.7150/jca.54163 (PMC8176422; doi:10.7150/jca.54163)
Supplement: Supplementary file 1 — Supplementary figure S1. [file jcav12p4277s1.pdf]

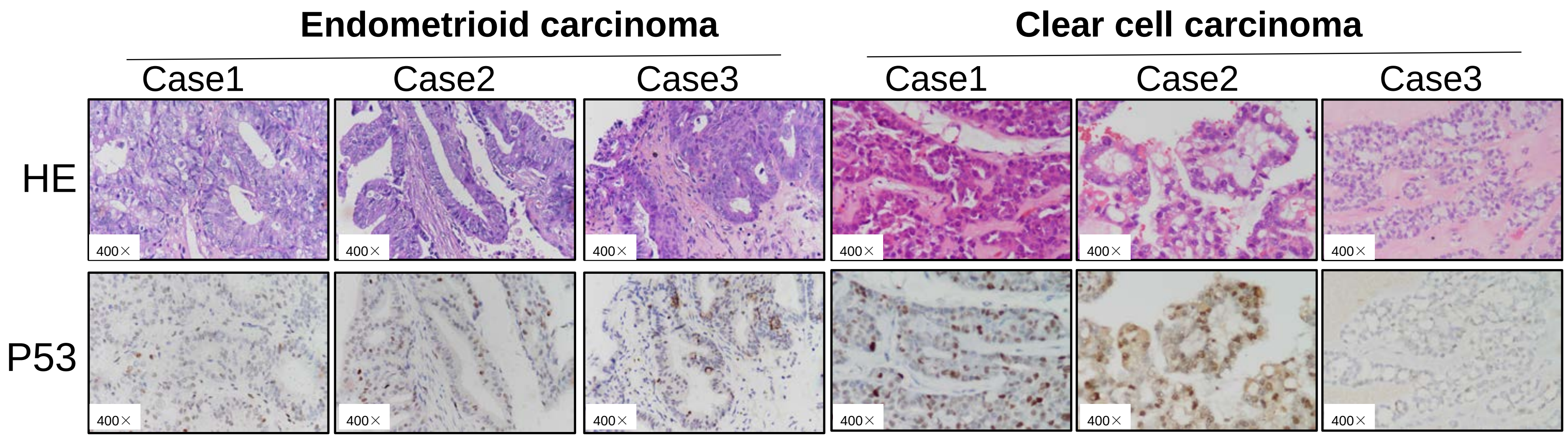

Figure S1. Immunohistochemical staining of p53 in endometrioid carcinoma tissues and clear cell carcinoma tissues.
